# Kv1.3 activity perturbs the homeostatic properties of astrocytes in glioma

**DOI:** 10.1038/s41598-018-25940-5

**Published:** 2018-05-16

**Authors:** Alfonso Grimaldi, Giuseppina D’Alessandro, Maria Amalia Di Castro, Clotilde Lauro, Vikrant Singh, Francesca Pagani, Luigi Sforna, Francesca Grassi, Silvia Di Angelantonio, Luigi Catacuzzeno, Heike Wulff, Cristina Limatola, Myriam Catalano

**Affiliations:** 1 Center for Life Nanoscience – Istituto Italiano di Tecnologia@Sapienza, Viale Regina Elena, 291-00185 Rome, Italy; 2 IRCCS Neuromed, Via Atinense 18, 86077 Pozzilli, IS Italy; 3 Department of Physiology and Pharmacology, Sapienza University, Piazzale Aldo Moro, 5-00185 Rome, Italy; 4 Department of Pharmacology, University of California Davis, 451 Health Sciences Drive, Davis, CA 95616 USA; 5 Department of Experimental Medicine, Section of Physiology and Biochemistry, University of Perugia, Piazzale Gambuli - 06123, Perugia, Italy; 6 Department of Chemistry Biology and Biotechnology, University of Perugia, Via Elce di sotto, 8 - 06123 Perugia, Italy; 7 Department of Physiology and Pharmacology, Sapienza University, Laboratory affiliated to Istituto Pasteur Italia – Fondazione Cenci Bolognetti, Piazzale Aldo Moro, 5-00185 Rome, Italy

## Abstract

Glial cells actively maintain the homeostasis of brain parenchyma, regulating neuronal excitability and preserving the physiological composition of the extracellular milieu. Under pathological conditions, some functions of glial cells could be compromised, exacerbating the neurotoxic processes. We investigated if the homeostatic activities of astrocytes and microglia could be modulated by the voltage-gated K^+^ channel Kv1.3. To this end we used *in vitro* and *in vivo* systems to model cell-to-cell interactions in tumoral conditions, using a specific inhibitor of Kv1.3 channels, 5-(4-phenoxybutoxy) psoralen (PAP-1). We demonstrated that PAP-1 increases astrocytic glutamate uptake, reduces glioma-induced neurotoxicity, and decreases microglial migration and phagocytosis. We also found in a tumor blood brain barrier model that Kv1.3 activity is required for its integrity. The crucial role of Kv1.3 channels as modulators of glial cell activity was confirmed in a mouse model of glioma, where PAP-1 treatment reduces tumor volume only in the presence of active glutamate transporters GLT-1. In the same mouse model, PAP-1 reduces astrogliosis and microglial infiltration. PAP-1 also reduces tumor cell invasion. All these findings point to Kv1.3 channels as potential targets to re-instruct glial cells toward their homeostatic functions, in the context of brain tumors.

## Introduction

Glial cells play pivotal roles in modulating cell-to-cell communication and the homeostasis of extracellular fluids in the CNS. Astrocytes are the main cell type involved in neurotransmitter re-uptake, modulation of synaptic function, and release of gliotransmitters. Astrocytes further directly interact with endothelial cell layers and control the integrity of the blood-brain barrier (BBB). Several types of K^+^ channels are involved in the modulation of intra and extracellular signaling pathways that regulate astrocyte activities in maintaining CNS homeostasis^1^. In addition to the inward rectifying Kir4.1 channel, important for the “spatial buffering” activity as third element of the tripartite synapses and for BBB integrity at the end feet facing the blood vessels^2^, astrocytes express TWIK1 and TREK1 channels, both implicated in the regulated gliotransmitter release^3^, and the large conductance Ca^2+^ activated K^+^ (BK) channels involved in the control of blood vessel tone^4^. Astrocytes also express several voltage-dependent K^+^ channels (Kv1.3, Kv1.4 and Kv1.5), but a clear understanding of their specific functions is presently lacking, likely due to their diffuse intracellular localization^5,6^.

Astrocytic uptake of extracellular glutamate through the excitatory amino acid transporter 2 (GLT-1) is driven by intra/extra cellular ion balance, being favoured by higher intracellular K^+^ concentrations^7–9^. In pathological microenvironments, such as those induced by epilepsy, neurodegenerative diseases or retinal inflammation, glial cells lose their ability to buffer glutamate^10^ and to balance the interstitial concentration of K^+^ ^11^. In brain tumors, astrocytes sustain glioma growth and invasion, exacerbating neuroinflammation^12^. In the early stages of glioma, astrocytes protect neurons by controlling the uptake of glutamate released by tumor cells^13^, however, as the tumor grows, astrocytes lose their protective role and, in fact, overexpression of GLT-1 prolongs survival in animal models of malignant glioma^14^.

Astrocyte end-feet control the expression of endothelial markers, such as the immunoglobulin-like cell surface glycoprotein HT7, and the angiotensin receptors 1 and 2^15^. In inflammatory conditions, such as those occurring in gliomas, astrocyte control is compromised, with the consequent leakage of endothelial tight junctions^16^ and disruption of the BBB^17^.

In brain tumors, soluble factors released by glioma-associated myeloid cells (GAMs; mainly macrophages and microglia), promote cell invasion and angiogenesis^18,19^. Different K^+^ channels expressed by GAMs modulate phagocytosis, cell migration and proliferation^20–22^, with Kv1.3 regulating microglia cell proliferation and neurotoxicity^23,24^. Kv1.3 channels are also expressed by transformed glia, with no correlation with tumor grade^25^, and their inhibition induces apoptosis in many human and mouse cancer cell lines^26,27^.

In this paper, we demonstrate that Kv1.3 channel inhibition modulates astrocyte and microglia reactivity in the context of glioma, reducing tumor growth and directly affects the invasive properties of glioma cells. All these findings indicate that Kv1.3 channels could serve as potential targets to rehabilitate glial cells, reducing the glioma-induced damage of surrounding brain parenchyma.

## Results

### Kv1.3 channels expressed by astrocytes mediate the neurotoxic effects of glioma-conditioned environment

**Figure 1:**
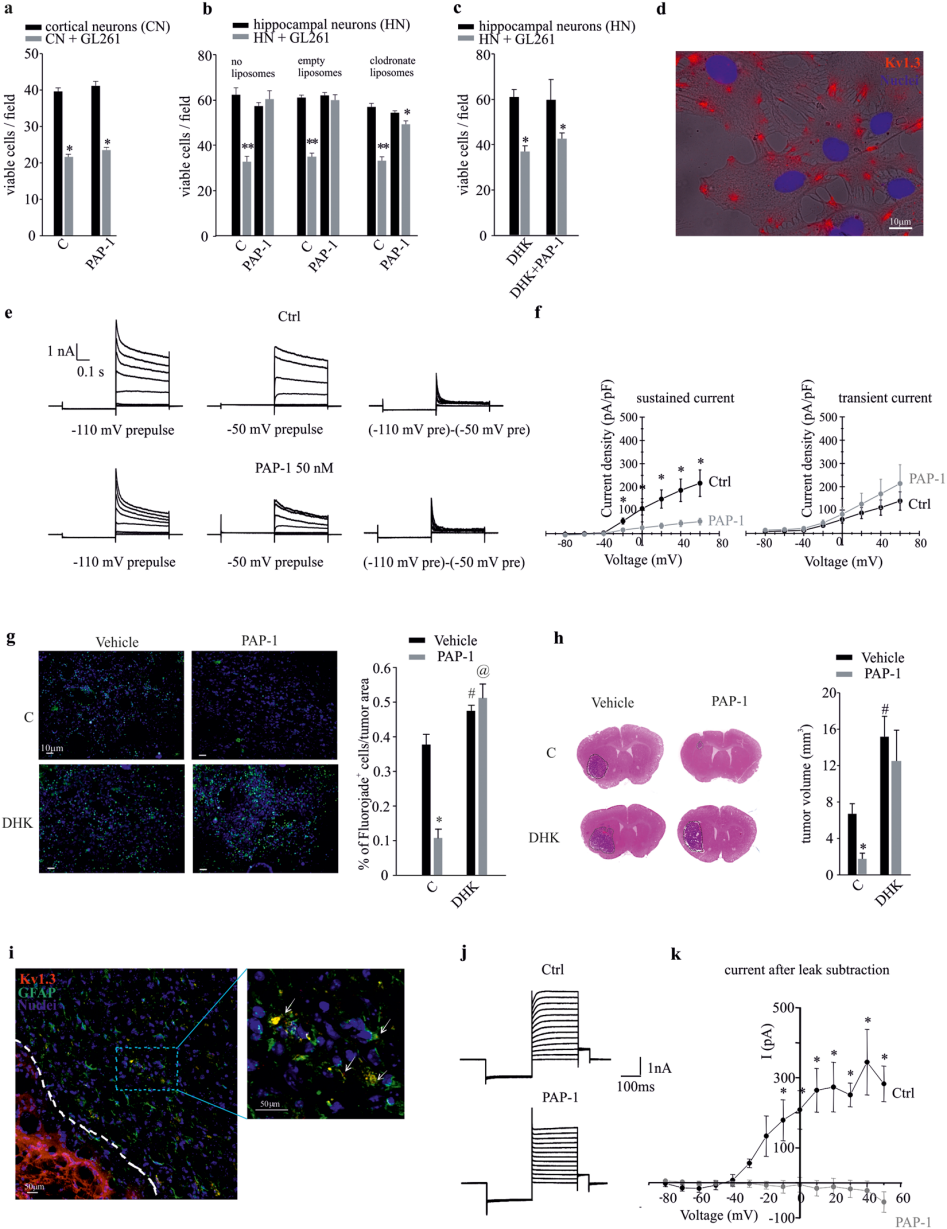
The inhibition of Kv1.3 channels induces neuroprotection against the toxic effects of glioma. (**a**) Cortical neurons (CN) co-cultured with GL261 cells (grey bars) or alone (black bars) were treated with PAP-1 (50 nM, 18 h) or vehicle (C) and analyzed for neuronal viability. Results are expressed as number of viable cells/field. *p = 0.001 vs C; n = 4, unpaired *t*-test. (**b**) Hippocampal neurons (HN) pre-treated or not with empty or clodronate-filled liposomes for 24 h, co-cultured as in (**a**) for a further 18 h in presence of PAP-1 (50 nM) or vehicle (C), were analyzed for neuronal viability. Results are expressed as number of viable cells/field. **p = 0.001 and *p < 0.05 vs C; n = 4, Holm-Sidak One Way ANOVA. (**c**) Hippocampal neurons (HN) co-cultured as in (**a**) were treated with PAP-1 or vehicle in the absence or presence of DHK (DHK + PAP-1) and were analyzed for neuronal viability. Results are expressed as number of viable cells/field. *p = 0.001 vs C; n = 4, Kruskal-Wallis One Way ANOVA. (**d**) Primary astrocytes were assayed by immunofluorescence for the membrane expression of Kv1.3 channels (red, Hoechst in blue) merged with bright field. (**e**) Representative families of current traces obtained in cultured astrocytes by applying voltage steps from −80 to +60 mV (delta = 20 mV) from a holding potential of −80 mV, preceded by a 400 ms prepulse at either −110 mV (left) or −50 mV (middle), under control conditions (black) and following bath application of 50 nM PAP-1 (grey). The current traces to the right were obtained by point-by-point subtraction of the currents recorded with the two different prepulses. (**f**) Mean current-voltage relationships obtained by applying the same voltage protocols described in (**e**) to 7 different astrocytes. The sustained current was taken from the end of the depolarizing pulses in the current traces obtained with the prepulse voltage of −50 mV. The transient current is the peak of the current traces obtained by point-by-point subtraction. *p < 0.001, paired *t*-test. (**g**) Neuronal death, measured as Fluoro-Jade staining (green; Hoechst in blue) in coronal brain slices of GL261-bearing mice, treated with vehicle or PAP-1 (40 mg/kg/die), in the absence or presence of DHK (10 mg/kg/die); right, immunofluorescence quantification expressed as % of Fluoro-Jade^+^ cell area normalized for tumor area, n = 3, *p = 0.001, ^#^p = 0.017, ^@^p = 0.005 vs Vehicle, Student-Newman-Keuls One Way ANOVA. (**h**) Tumor volumes in the brain of mice treated as in (**g**),with or without DHK (10 mg/kg/die). Right, mean of tumor volumes (in mm^3^) ± s.e.m., n = 8, *p = 0.013, ^#^p = 0.018 vs Vehicle, Kruskal-Wallis One Way ANOVA. (**i**) Immunofluorescence analyses of Kv1.3 expression (red) on GFAP-positive cells (green) in coronal brain slices of GL261-bearing mice in peritumoral region; in the magnification (right) arrows indicate co-expression of Kv1.3 and GFAP in the same cell. (**j**) Representative current traces obtained by whole-cell patch-clamp recordings from peritumoral astrocytes in GL261-bearing mice. Voltage steps were applied as in (**e**) with a −110 mV prepulse, under control conditions (Ctrl) and following bath application of 100 nM PAP-1. (**k**) Mean current-voltage relationships were determined subtracting the steady-state current from the peak current at each voltage step, before (Ctrl, in black) and after PAP-1 application (in grey). The resulting current (I) was expressed in pA. PAP-1 treatment abolished the voltage-activated current observed in Ctrl condition, n = 4, *p < 0.05, paired *t*-test.

In the microenvironment of glioma-infiltrated brain, glutamate released by tumor cells is the main mediator of neuronal cell death^28^ and hyperexcitability^29–31^. Glial cells are actively involved in glutamate clearance from the extracellular space, but this activity is deeply unbalanced by glioma-released factors^29^. To verify the hypothesis that Kv1.3 channel activity could modulate the ability of glial cells to prevent glioma-induced neurotoxicity, we co-cultured primary murine cortical neurons with GL261 glioma cells in the presence of the Kv1.3 channel inhibitor PAP-1 (50 nM). Under these conditions, we observed that the co-incubation with glioma cells significantly reduced neuronal viability, and that PAP-1 treatment was not able to prevent neuronal death (Fig. 1a). Primary cultures of cortical neurons, however, contain only few “contaminating” glial cells^32^; to reproduce a more physiological microenvironment, the same experiment was performed by co-culturing GL261 cells with murine hippocampal neurons, containing as much as 40% glial cells^33^. In this neuronal/glial mixed population, PAP-1 treatment efficiently reduced glioma-induced neurotoxicity (Fig. 1b). To evaluate the relative contribution of astrocytes and microglia, the experiment was performed in the presence of clodronate-containing or empty (control) liposomes. Preliminary experiments demonstrate that clodronate eliminates 99.6 ± 0.2% of microglia in 48 h^33^. Under these conditions, PAP-1 was still able to exert neuroprotection against glioma-induced toxicity (Fig. 1b), although to a reduced extent, as the number of viable cells remained slightly but significantly reduced. This demonstrates the primary involvement of astrocytes in the PAP-1 mediated effects together with possible minor contribution by microglia. To investigate whether this astrocyte-dependent neuroprotection involved the activity of glutamate transporters, the experiments were performed in the presence of dihydrokainic acid (DHK, 500 μM, 18 h), a specific inhibitor of GLT-1. The results obtained show that Kv1.3 inhibition with PAP-1 was not able to prevent neuronal cell death induced by GL261 glioma cells in the presence of DHK (Fig. 1c). Of note, to exclude the possibility that the lack of PAP-1’s neuroprotection in presence of DHK could be due to a depolarizing effect of the GLT-1 inhibitor, we measured astrocyte resting potential upon DHK application (500 μM) and did not observe depolarizing effects (data not shown).

By immunofluorescence microscopy, we observed that, *in vitro*, murine astrocytes express Kv1.3 channels on the plasma membrane (Fig. 1d), unlike the main intracellular localization reported in rat cultured astrocytes^5^. Accordingly, we found evidence for the functional expression of this channel in primary astrocyte cultures: as previously shown^34,35^, cultured astrocytes expressed both transient and sustained voltage-gated K^+^ currents, that can be isolated by depolarizing pulses preceded by different pre-pulse voltages (Fig. 1e). Notably, a PAP-1 concentration of 50 nM inhibited the sustained component, while leaving the transient component unaltered (Fig. 1f and Supplementary Fig. 1a). Inhibition of the sustained current by PAP-1 was mostly complete at 100 nM (Supplementary Fig. 1b).

To investigate whether a neuroprotective effect could also be observed *in vivo*, C57BL/6N mice were injected with syngeneic GL261 glioma cells and treated with PAP-1 (i.p. 40 mg/kg/die, starting 7 days after tumor injection) for 10 days. Total brain concentrations of PAP-1, evaluated by HPLC/MS analysis at 2 and 12 h after the last i.p. administration on day-10 were, respectively, 4.49 ± 2.28 μM and 5.37 ± 1.96 μM (n = 3 animals per time point), demonstrating that sufficient concentrations of PAP-1 where present to exert pharmacological effects considering PAP-1’s protein binding of 98%. The data in Figure 1g show that PAP-1 treatment reduced neuronal death in the tumor region, evaluated by Fluoro-Jade staining, compared to vehicle-treated mice. Notably, PAP-1-induced neuroprotection was abolished by simultaneous DHK administration (10 mg/kg/die i.p.), indicating that the PAP-1 effects involve the active participation of the glutamate transporter GLT-1. Accordingly, PAP-1-treated mice had reduced tumor volume (Fig. 1h, top), an effect abolished by DHK administration (10 mg/kg/die i.p.). DHK treatment increased tumor volume *per se*, as expected considering the key role of glutamate-induced neurotoxicity in glioma expansion (Fig. 1h, bottom), differently to what we observed *in vitro* (Fig. 1c), possibly due to the heterogeneity between the *in vivo* and *in vitro* systems. These data confirm the key role of glutamate transporter activity in PAP-1 mediated neuroprotection against glioma.

By immunofluorescence analysis, performed on brain slices obtained from GL261-bearing mouse, we confirmed the expression of Kv1.3 channels on GFAP-positive cells (astrocytes) present in the peritumoral area (Fig. 1i, arrows). To evaluate the functionality of Kv1.3 channels in peritumoral astrocytes, patch-clamp recordings were performed. All astrocytes displayed passive membrane properties with a hyperpolarized membrane potential (−81.89 ± 3.05 mV, n = 11), small membrane capacitance (27.45 ± 2.87 pF, n = 16) and low membrane resistance (17.19 ± 1.26 MOhm, n = 16). The majority of astrocytes examined under voltage-clamp mode showed large passive currents with a linear current-voltage relationship. In a proportion of all tested astrocytes (~30%) we also observed a slowly activating voltage-dependent current which was blocked by PAP-1 application (Fig. 1j,k). Membrane potential (V_m_) and membrane resistance (R_i_) were not affected by PAP-1 treatment (V_m_: Ctrl = −84.25 ± 0.07 mV, PAP-1 = −86.96 ± 0.03 mV, n = 8, paired Student’s *t*-test p = 0.52; R_i_ Ctrl = 26.38 ± 7.74 mV, PAP-1 = 20.63 ± 5.63 mV, n = 5 paired Student’s *t*-test: p = 0.56). These data confirm the presence of Kv1.3 on astrocytes, at least in the peritumoral regions.

### Kv1.3 inhibition enhances astrocyte glutamate buffering

**Figure 2:**
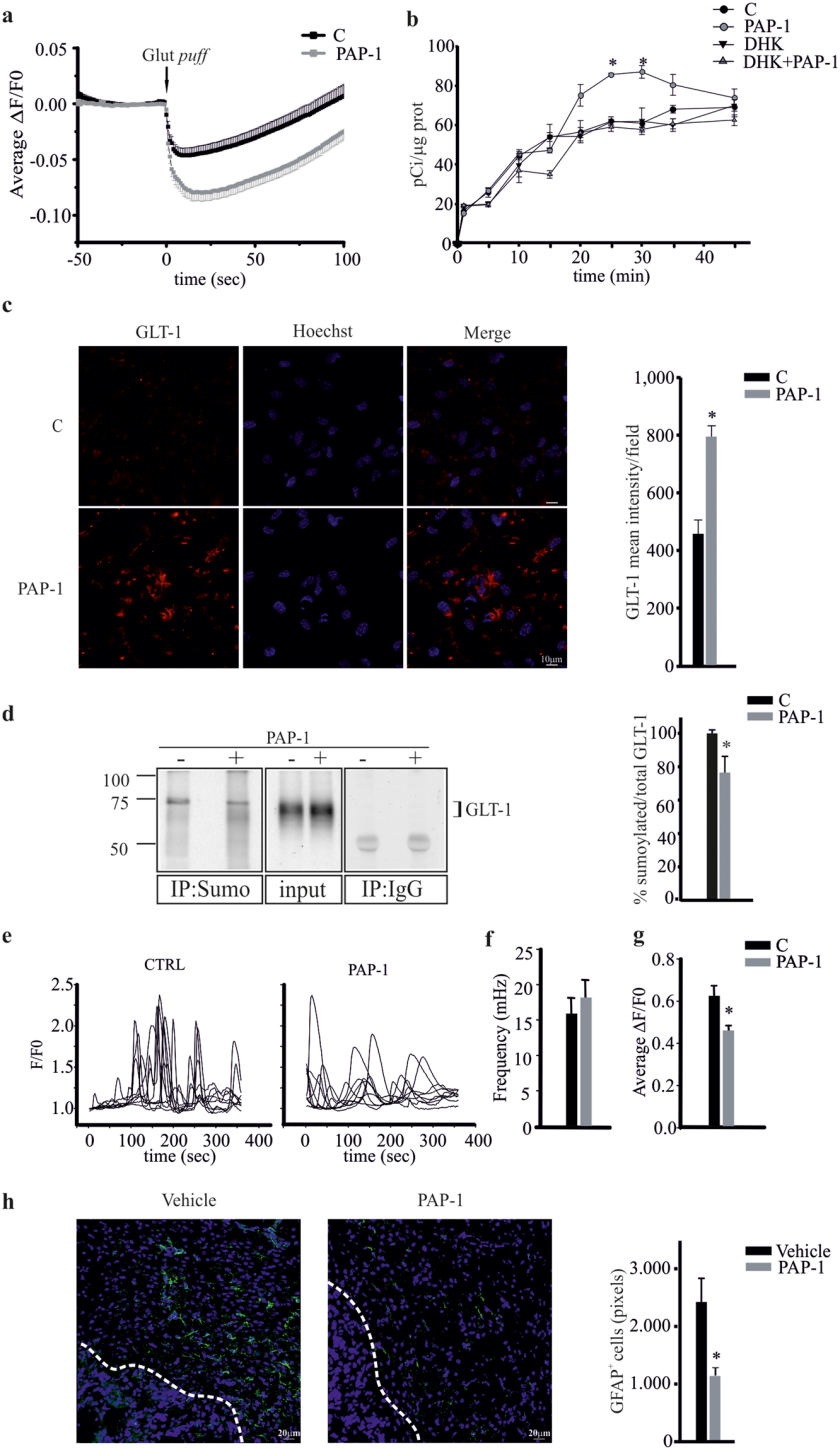
Kv1.3 activity modulates glutamate buffering on astrocytes. (**a**) Time course of fluorescence ratio (ΔF/F0) changes induced by a puff of glutamate (1 mM for 0.5 sec, Glut *puff*) onto astrocytic cultures loaded with BCECF-AM (10 μM, 45 min) and pre-treated with vehicle (n = 71) or PAP-1 (100 nM, n = 69). At peak ΔF/F0 in PAP1 = −0.08 ± 0.008 *vs* −0.046 ± 0.014 in Ctrl p = 0.0009, unpaired Student’s *t*-test). **(b)** Astrocytes were treated with PAP-1 (50 nM, grey circles) or not (black circles) with or without DHK (500 μM, triangles) for different times (from 2 to 45 min) and analyzed for intracellular D-[^3^H]Asp, as described in the Methods section. Results are expressed as pCi/μg proteins and are the mean ± s.e.m. of at least 5 triplicate experiments. *p = 0.001 *vs* C of the correspondent time point, Holm-Sidak method One Way ANOVA. (**c**) Confocal images of astrocytes, untreated (C) or treated with PAP-1 (50 nM, 25 min), stained for plasma membrane GLT-1 (red, Hoechst in blue), scale bar 10 μm. On the right, data represent the mean fluorescence intensity of red signals per field ± s.e.m. n = 4, *p = 0.042 *vs* C, unpaired *t*-test. (**d**) Astrocytes untreated (−) or treated (+) with PAP-1 (50 nM, 25 min) were immunoprecipitated for Sumo-1 or control IgG and immmuno-blotted for GLT-1; total lysate (input) is shown. On the right, data represent the mean ± s.e.m. of optical density of sumoylated GLT-1 expressed as % of the input, *p = 0.028 *vs* C, unpaired *t*-test. (**e**) Representative time course of spontaneous Ca^2+^ oscillation (F/F0) in cultured astrocytes loaded with Fluo4-AM in CTRL (left panel) and after PAP-1 application (right panel). Each trace in the panel represent a single ROI in the field. (**f**) Quantification of Ca^2+^ transients before and after PAP-1 treatment. (**g**) Average ΔF/F0 of Ca^2+^ transient in CTRL condition and after PAP-1 application. (**h**) Coronal brain sections of GL261-bearing mice treated with vehicle or PAP-1 (40 mg/kg/die) were stained for GFAP (green; Hoechst, in blue) and visualized at the border of the tumor (white dashed line), scale bar 20 μm. Right, data represent the mean area (in pixels) covered by GFAP^+^ cells present at a distance up to 100 μm from the tumor border (mean ± s.e.m. n = 6, *p = 0.015, unpaired *t*-test).

To directly confirm the link between Kv1.3 channels and glutamate transporter activity, we investigated the intracellular pH changes induced by PAP-1 (100 nM) treatment, taking advantage of glutamate/proton co-transport. Loading cultured murine astrocytes with the pH-sensitive dye BCECF-AM, we tracked, in real-time, the intracellular pH changes induced by glutamate application, in control conditions and in the presence of PAP-1. Figure 2 a shows a decrease in cell fluorescence, corresponding to increased intracellular [H^+^], upon puffing glutamate (1 mM). The decrease in BCECF fluorescence was more pronounced in PAP-1 treated astrocytes indicating that the inhibition of Kv1.3 channels enhanced glutamate transporter activity. We also directly measured the effect of PAP-1 on glutamate transport in astrocytes: as reported in Figure 2b, astrocytes increased [^3^H]-Asp uptake upon PAP-1 (50 nM) application, and DHK (500 μM) inhibited this effect, as expected for a GLT-1-mediated mechanism. We then investigated the effect of PAP-1 treatment on GLT-1 expression on cultured astrocytes: confocal immunofluorescence analysis with a C-terminal specific GLT-1 antibody demonstrated increased expression of GLT-1 protein upon PAP-1 (50 nM) treatment (Fig. 2c). GLT-1 membrane trafficking is regulated by Ca^2+^ mediated intracellular pathways^36^ that lead to post-translational modifications of the transporter^37^. One of the ubiquitin like molecules is SUMO-1 (Small Ubiquitin MOdifier 1) that controls GLT-1 transport to the plasma membrane^38^: we measured the levels of sumoylated GLT-1 (which represents the intracellular form of the transporter) by immunoprecipitation experiments. The data in Figure 2d show that anti-Sumo-1 Ab also co-immunoprecipitated GLT-1, and this association was significantly reduced in PAP-1 treated astrocytes, thus demonstrating that Kv1.3 inhibition induced a decrease of the intracellular form.

To investigate if intracellular Ca^2+^ levels were affected by Kv1.3 blockade we performed calcium imaging experiments on primary astrocytes. Results show that upon PAP-1 (100 nM) treatment, the amplitude, but not the frequency, of spontaneous calcium oscillations was reduced (Fig. 2e,f,g).

Additionally, since it has been reported that glutamate transporter expression in astrocytes inversely correlates with the expression of glial fibrillary acidic protein GFAP^39^, which is known to be strongly increased in brain parenchyma surrounding glioma^12^, we investigated whether Kv1.3 blockade affected GFAP expression in our glioma mouse model. In accordance, we found that PAP-1 treatment in glioma-bearing mice significantly decreased peritumoral staining with GFAP (Fig. 2h), confirming that the increased glutamate transporter activity induced by Kv1.3 inhibition correlates with the reduction of astrogliosis.

### Kv1.3 activity modulates the blood brain barrier (BBB) integrity

**Figure 3:**
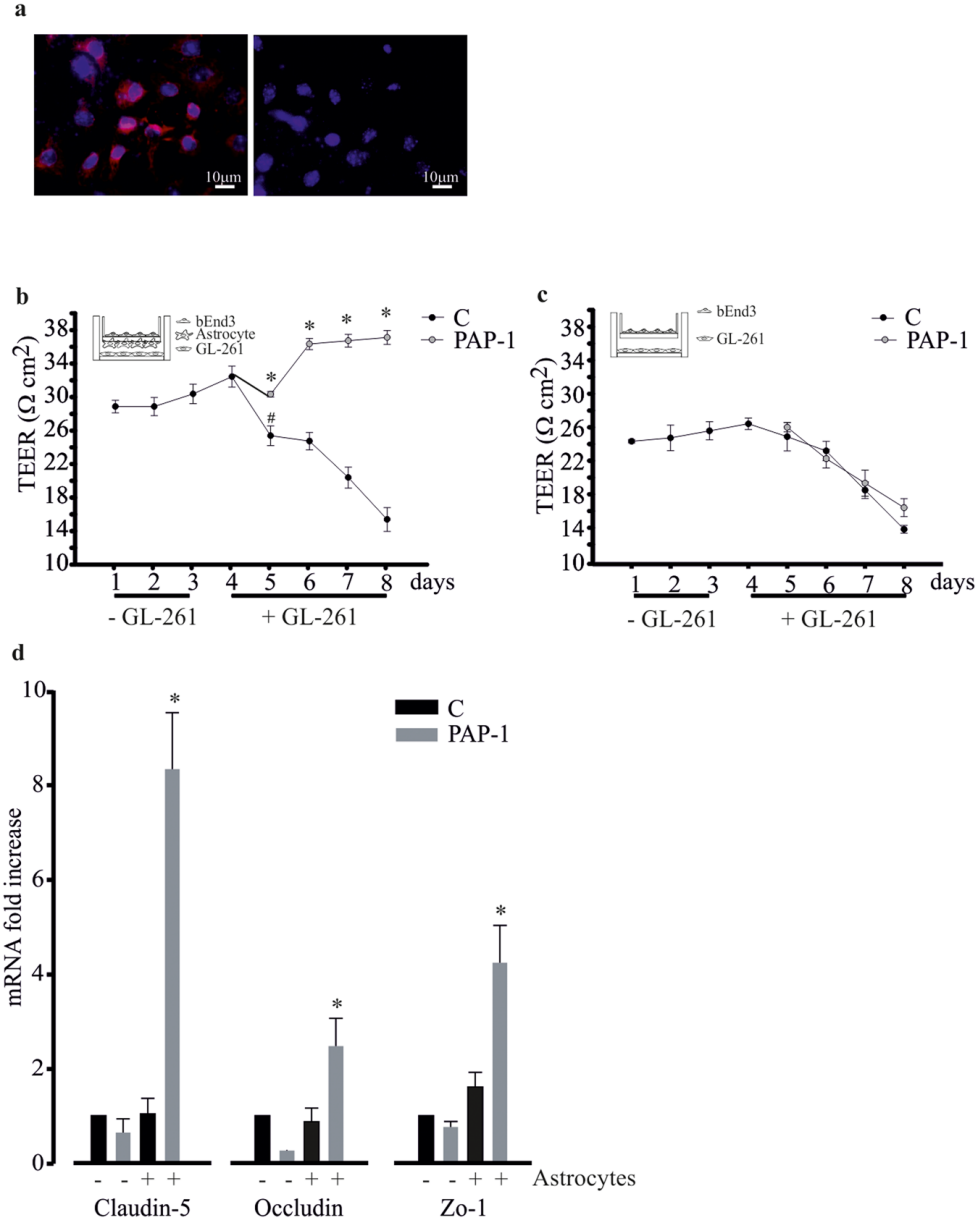
Kv1.3 channel activity controls tight junction protein expression on bEnd.3. (**a**) Immunofluorescence analysis of Kv1.3 (in red, Hoechst in blue) on endothelial bEnd.3 cells. On the right, cells stained only with secondary Ab as control. (**b**) Astrocytes were co-cultured with bEnd.3 in the absence (−GL261) or presence (+GL261) of GL261 cells (as depicted in the inset), and treated or not with PAP-1 (50 nM). Trans-endothelial electric resistance (TEER, in Ωcm2) was measured at the indicated time points. (**c**) bEnd.3 in the absence (−GL261) or presence (+GL261) of GL261 cells (as depicted in the inset), and treated or not with PAP-1 (50 nM) were assayed for TEER (in Ωcm^2^) at the indicated time points. (**d**) RT-PCR gene expression of claudin-5, occludin and zo-1 in untreated (C) or PAP-1 (50 nM) treated bEnd.3 co-cultured or not with astrocytes. Data are expressed as fold increase in co-cultures vs bEnd.3 alone (no astrocytes) and are the mean ± s.e.m., n = 4, *p = 0.001, Dunn’s method One Way ANOVA.

One form of brain damage induced by glioma is disruption of the BBB^40^; we therefore investigated the effect of Kv1.3 inhibition on BBB integrity, also considering that Kv1.3 could be present on endothelial cells^41^. To this end, we used an *in vitro* model of the BBB composed of the murine endothelial cells bEnd.3 (expressing Kv1.3, Fig. 3a) and primary astrocytes. This co-culture system creates a barrier that reaches a trans-endothelial electrical resistance (TEER) of about 30 Ωcm^2^ in 24 h and remains stable up to 96 h. In the presence of GL261 cells, the TEER value falls to 25 Ωcm^2^ after 1 day, reaching about 15 Ωcm^2^ after an additional 3 days. In the presence of PAP-1 (50 nM) this effect was not present, indicating that Kv1.3 inhibition counteracts the deteriorating effect of glioma-released factors on BBB integrity (Fig. 3b). The inhibition of Kv1.3 channel, however, cannot prevent the effect of glioma cells on BBB resistance when the barrier only consisted of endothelial cells (Fig. 3c), demonstrating that the significant effect of PAP-1 is on astrocytes. To exclude that the PAP-1 effects were mediated by changes in gap junction coupling efficacy, we performed dye-coupling experiments in astrocytic cultures, loading a single cell with the membrane-impermeant dye Alexa Fluor 488 (40 μM) via the patch pipette. The analysis of dye spreading was performed by counting the number of stained cells/fields 10 min after establishing whole-cell recordings. We did not observe differences in the percentage of stained cells between control and PAP-1- (100 nM) treated cultures (CTRL: 95.83 ± 4.17% n = 4; PAP-1: 85.71 ± 10.102%, n = 4, unpaired t-test = 0.39). This suggests that the Kv1.3 inhibitor PAP-1 does not alter gap junctional coupling among astrocytes. Since astrocytes modulate the expression of tight junctions on endothelial cells^42^, we analysed the effect of Kv1.3 inhibition on the mRNA expression of specific proteins that compose the tight junction complex, such as claudin-5, occludin and zonula occludens 1 (Zo-1), in the presence and in the absence of astrocytes (cultured as shown in insets Fig. 3b,c). The data in Figure 3d show that Kv1.3 inhibition significantly increased the expression of tight junction proteins on endothelial cells only when co-cultured with astrocytes, confirming the key role of astrocytes in controlling BBB integrity, through the modulation of Kv1.3 activity.

### Glia-mediated neuroprotective effect of PAP-1 against glioma is partially dependent on microglial cells

**Figure 4:**
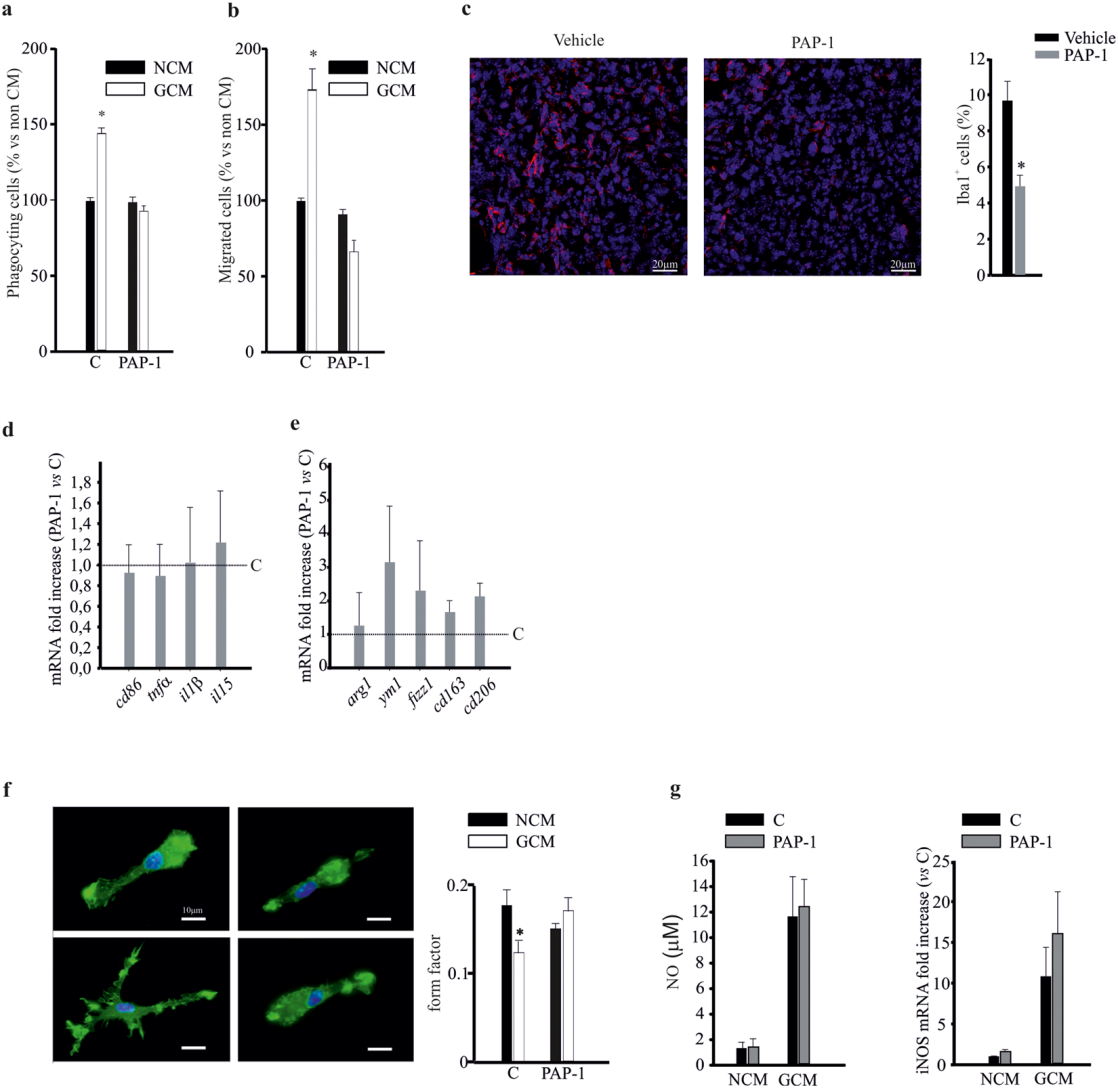
Kv1.3 activity modulates microglia functions. (**a**–**b**) Non-conditioned medium (NCM)- and glioma conditioned medium (GCM)-treated microglia, in the absence (C) or presence of PAP-1 (50 nM) assayed for phagocytosis (**a**) and migration (**b**). Data are expressed as the % of phagocytosing (**a**) and migrated (**b**) cells ± s.e.m. *p = 0.001vs NCM; n = 4, Kruskal-Wallis One Way ANOVA on Ranks. (**c**) Coronal brain sections of GL261-bearing mice treated with PAP-1 (40 mg/kg/die) or vehicle were stained for Iba1 (red) and Hoechst (blue), scale bar 20 µm. On the right, % of Iba1^+^ cell area normalized for tumor area, *p = 0.002, unpaired *t*-test, n = 6. (**d**,**e**) RT-PCR for pro- (*cd86, tnfα, il1α, il15*) and anti- (*arg1, ym1, cd163, cd206*) inflammatory genes expressed by CD11b^+^ cells extracted from ipsilateral hemisphere of brains of GL261-bearing mice treated with vehicle (C) or PAP-1 (40 mg/kg/die). Data are expressed as fold change of PAP-1-treated *vs* vehicle-treated samples (C, dashed lines) and are the mean ± s.e.m., *p < 0.05 by Student’s *t*-test; n = 4. (**f**) NCM- and GCM-treated microglia in the absence (C) or presence of PAP-1 (50 nM), stained with phalloidin (green) and Hoechst (blue), scale bar: 20 μm. Form factor values (calculated as reported in the Methods section) are shown in the graph (right) and are the mean ± s.e.m. of n = 4, *p = 0.001; Kruskal-Wallis One Way ANOVA. (**g**) NCM- and GCM-treated microglia in the absence (C) or presence of PAP-1 (50 nM) were assayed for NO production (expressed in µM) and *iNOS* mRNA expression (expressed as fold increase), n = 4, there is not a statistically significant difference between C and PAP-1 groups both on NCM and GCM conditions (p = 0.082 for NO production; p = 0.088 for *iNos* mRNA expression).

In the brain, Kv1.3 channels are also functionally expressed by microglial cells^20,22^, and, as we reported in Figure1b, microglia are partially involved in PAP-1-mediated neuroprotection against glioma. We investigated whether the inhibition of Kv1.3 channels affects microglial functions such as phagocytosis and cell migration, upon treatment with GL261-conditioned medium (GCM). The results shown in Figure 4a,b demonstrate that PAP-1 treatment completely abolished the GCM-induced increase of phagocytosis and migration of microglial cells. Interestingly, we report similar effects *in vivo*: when glioma-bearing mice were treated with PAP-1 as described above, infiltration of the tumor mass by microglia and other myeloid (Iba-1^+^) cells was markedly reduced (Fig. 4c), confirming the crucial role of Kv1.3 in mediating glioma-induced myeloid cell migration. Of note, CD11b^+^ cells, isolated from the brains of these animals, did not show any phenotype change, as demonstrated by the RT-PCR expression analysis of pro-inflammatory (*cd86, tnfα*, *il1α, il15*; Fig. 4d) and anti-inflammatory (*arg1, fizz1, ym1, cd163, cd206*¸ Fig. 4e) genes. We also analysed the modification of cell shape of microglia exposed to glioma, by measuring the “form factor” (see Methods). We observed that microglia exposed to GCM acquired a ramified shape, not observed upon PAP-1 treatment (Fig. 4f). PAP-1 treatment did not affect the viability of cultured microglia at 24 and 48 h (data not shown). It is known that in the presence of glioma, microglia release neurotoxic amounts of nitric oxide (NO)^28^. We confirmed this effect, together with an increased expression of iNOS by microglia in our co-culture conditions, and observed that Kv1.3 inhibition altered neither NO release, nor iNOS expression (Fig. 4g). These results indicate that Kv1.3 activity on microglia does not mediate glioma-stimulated NO production.

### Kv1.3 inhibition decreases glioma cell migration

**Figure 5:**
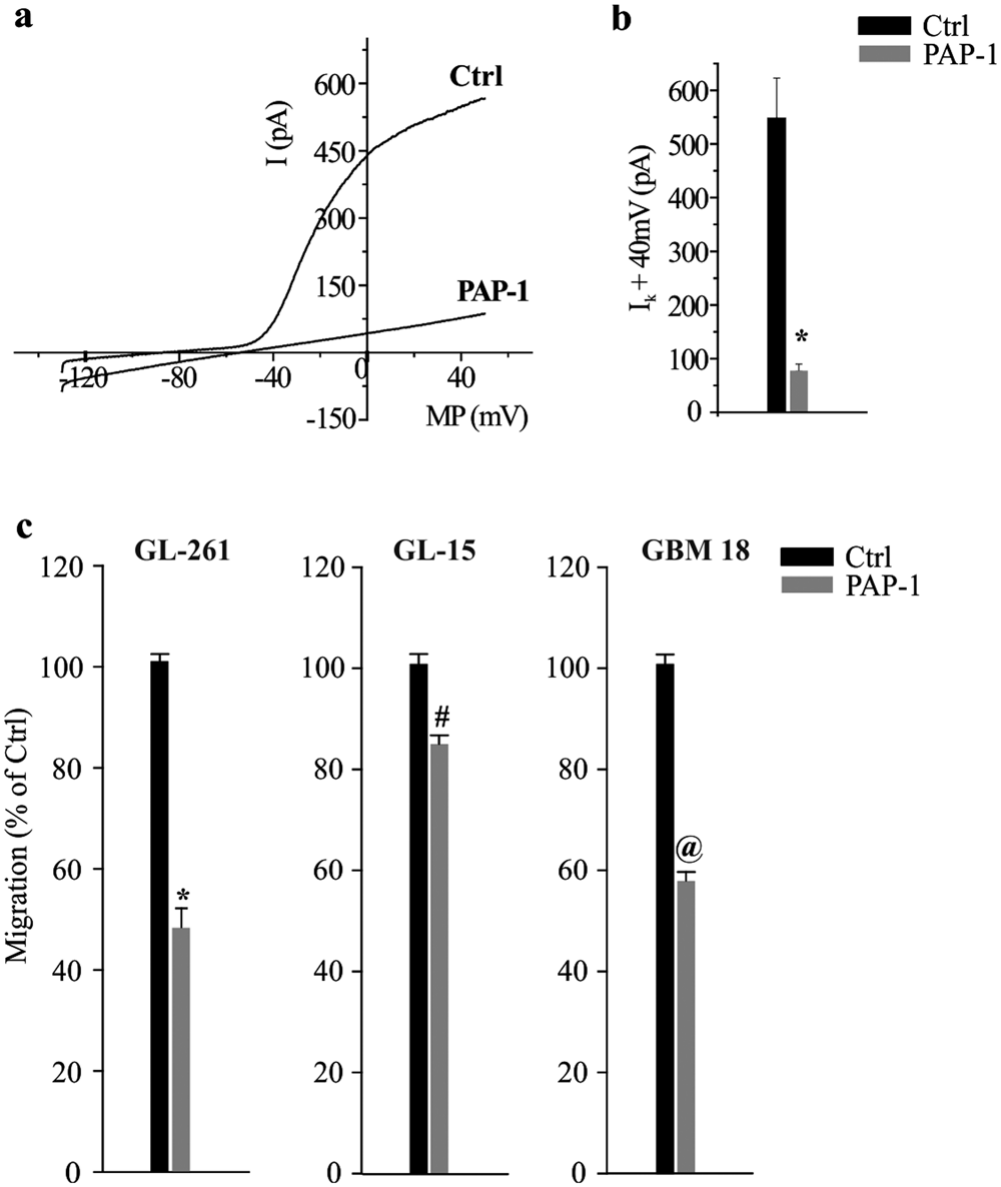
Kv1.3 is expressed by glioma cells and modulates their migration. (**a**) Typical current traces in response to repeated voltage ramps from −120 to +50 mV (holding potential −70 mV) in Ctrl and PAP-1 (100 nM) treated GL261 cells. (**b**) Bar graph representing PAP-1 sensitive current amplitude in GL261 cells, n = 14, *p = 0.01, *t*-test. (**c**) Migration assay on untreated (Ctrl) and PAP-1 (50 nM, 4 h) treated GL261, GL-15 and GBM18 cells; data are the mean ± s.e.m., n = 4, *p = 0.001, ^#^p = 0.05, ^@^p = 0.001, unpaired *t*-test.

Kv1.3 channels are expressed by glioma cells^25^ and specifically by GL261 cells (Fig. 1i). We confirmed their functional expression on GL261 cells by recording K^+^ currents that were inhibited by PAP-1 treatment (Fig. 5a,b). We then investigated the involvement of Kv1.3 channels in glioma migration. To this end, we measured the effect of PAP-1 (50 nM) on basal cell migration in murine GL261, and in human GL15 and GBM18 (primary glioblastoma from a patient) cells. In the three cell types, we observed a significant reduction of migration rate when Kv1.3 channels were inhibited (Fig. 5c). As already reported for other tumor cells, the inhibition of cell migration positively correlates with the block of Regulatory Volume Decrease (RVD)^43^. We treated GL261 cells with a hypo-osmotic shock to induce a rapid increase of cell volume. Under control conditions the RVD was almost complete in about 20–25 min while, in the presence of PAP-1, RDV was significantly reduced (Supplementary Fig. 2). The inhibition of Kv1.3 channels with PAP-1 in GL261 line did not interfere with cell proliferation or with cell cycle (data not shown), as recently reported on the same cells^6^. Taken together, these data support the hypothesis that Kv1.3 activity contributes to tumor cell migration and invasion of brain parenchyma through modulation of cell volume.

## Discussion

Kv1.3 channels, members of the Shaker family of voltage-gated K^+^ channels^44^, are involved in metabolic control^45^ and widely expressed in the immune system, where they control Ca^2+^ signalling in T cells, B cells and innate immune cells^46^. Kv1.3 is also found in the CNS, especially in neurons of the olfactory system^47^, in glial cells such as microglia^23^ and astrocytes^48^, and in gliomas^25^. Based on this expression pattern, we considered it of interest to investigate the role of Kv1.3 in the cross talk between glioma and cells of the brain parenchyma.

In this paper, we demonstrated that Kv1.3 channels, which are expressed in astrocytes in the glioma environment, modulate GLT-1 trafficking and functions: in particular, Kv1.3 inhibition decreases the desumoylation of the GLT-1 transporter, thereby promoting its membrane expression and, consequently, its activity. Moreover, as reported for lymphocytes^46^, Kv1.3 channel inhibition induces a reduction of spontaneous Ca^2+^ oscillations in astrocytes, demonstrating that Kv1.3 activity modulates the intracellular calcium homeostasis. This effect could reduce Ca^2+^-dependent mechanisms, for instance regulating post-translational modifications of GLT-1 that govern membrane trafficking of the transporter^36,37^. The modulation of GLT-1 activity represents the mechanism used by Kv1.3 to induce neuroprotection against glioma-induced excitotoxicity. This was demonstrated *in vitro*, in a mixed neuron/glia population co-cultured with glioma, and *in vivo*, in glioma bearing mice, where PAP-1 treatment reduced neuronal cell death. In these conditions, GLT-1 inhibition with DHK completely abolished the neuroprotective effects of PAP-1, thus demonstrating a link between Kv1.3 activity and glutamate transport. In addition, glutamate uptake is known to be regulated by the increase of extracellular K^+^ over the physiological range^9,49^. Consequently, the inhibition of Kv1.3 channels by PAP-1 could modulate the clearance of glutamate by influencing K^+^ concentrations.

Kv1.3 inhibition also reduces glioma-induced astrogliosis, in accordance with findings in rats with autoimmune optic neuritis, where GFAP expression in the regions of the optical nerve was reduced upon PAP-1 treatment^50^. GFAP overexpression is also inversely correlated with GLT-1 expression^51^, and a reduced GLT-1 level is considered a marker of “reactive” astrocytes both in the hippocampus and in the cerebral cortex^52^.

Astrocytes are component and active players in the BBB formation and maintenance, also modulating differentiation, pinocytosis and fenestrations of endothelial cells^53^. Using an *in vitro* BBB model, composed of endothelial cells and astrocytes, we demonstrated that Kv1.3 inhibition reduced glioma-induced BBB disruption, and increased the expression of tight junction proteins on endothelial cells. This effect is mediated by astrocytes, because it was not observed when only endothelial cells were treated with PAP-1. This result confirms the importance of a correct electrolytic equilibrium in the regulation of BBB integrity^54^.

We demonstrated that astrocytes express a voltage-gated, sustained outward current highly sensitive to low concentrations of PAP-1, suggesting the expression of Kv1.3 channels on the plasma membrane. Previous data obtained in rat astrocytes, suggested a *cis*-Golgi and plasma membrane expression of Kv1.3 subunits^5,55^. Our data add a new finding, demonstrating that, in pathological brain conditions, some Kv1.3 can be functionally expressed at the cell surface also of astrocytes. Although astrocytes have a hyperpolarized membrane potential, together with low membrane resistance, which basically prevents strong cell depolarization, we suggest that Kv1.3 channels are activated locally, at the peri-synaptic processes which can “sense” much stronger depolarizations compared to the soma, due to the high surface-to-volume ratio (SVR)^56,57^. However, we must consider that the concentration of PAP-1 we used (50 nM), in addition to blocking Kv1.3 (EC_50_ = 2 nM), can also affect other Kv1 channels such as Kv1.5 (EC_50_ = 45 nM), and Kv1.6 (EC_50_ = 62 nM)^58^, both contributing to the voltage-gated K^+^ current^34^. However, our best fit parameters with astrocytes were Kd = 23.8 nM, n_H_ = 1.09. Since for pure Kv1.3 we get a n_H_ of 2, which is also in keeping with the binding site that fits 2 PAP-1 molecules, this could indicate the presence of hetero-multimers. Further experiments with more selective inhibitors are needed to conclusively clarify this point.

The expression and functional importance of Kv1.3 on microglia has been previously repeatedly shown^20,22^. However, we observed that the inhibitory effect of Kv1.3 blockade on glioma-induced neurotoxicity was essentially maintained when microglia were depleted, indicating a marginal role of these cells in this context. We demonstrated that Kv1.3 inhibition reduced the ability of GAM to phagocytose, differently than in a LPS-induced model of inflammation^58^, demonstrating the high plasticity of microglia exposed to different stimuli. In microglia, Kv1.3 blockade also reduced migration in response to glioma. This was shown *in vitro*, in cultured microglia, where PAP-1 treatment reduced cell movement towards glioma-released soluble factors, and *in vivo*, in glioma-bearing mice, where PAP-1 treatment reduced the extent of GAMs infiltration in tumor mass. These data support the current view that the inflammatory status of microglia is correlated with the activation level of several K^+^ channels such as the inward rectifying Kir2.1, the Ca^2+^ activated KCa3.1, KCa2.3, KCa1.1, and the voltage-dependent Kv1.3, Kv1.1, Kv1.5, Kv3.1^22^. These channels are differently involved in inflammation (depending on the type of stimuli), by contributing to modify microglial shape, volume and gene expression, and by affecting cell motility^20,22,23,59^. In contrast to KCa3.1 inhibition^20^, Kv1.3 blockade was not able to change the phenotype of GAMs, indicating that the inhibition of this channel operates as a functional modification of GAMs (that became less motile, Fig. 4c) without interfering with their expression level of inflammatory genes, at least in the context of gliomas. Kv1.3 channels are also highly expressed by T lymphocytes, where they modulate different cellular activation states^60,61^. In head and neck tumors, Kv1.3 is poorly expressed by infiltrating T cells^62^; while the overexpression of Kv1.3 in melanoma-activated T cells increases the anti-tumor function of these cells^63^. Further studies will be necessary to define the effect of Kv1.3 inhibition in the T cells that infiltrate glioma.

Kv1.3 is over-expressed in many tumors (breast and colon cancer, leiomyosarcoma, alveolar rhabdomyosarcoma, lymph node cancer, neoplastic B cells)^26,27^ including GBM^25^, and we confirmed the presence of Kv1.3 channels and PAP-1 sensitive currents on GL261 cells. We demonstrated that Kv1.3 inhibition strongly reduced the ability of GL261 cells to migrate, possibly due to effects on cell volume changes, as also shown for other cell types^64^.

Even if we focused our current study on the role of astrocytes in the antitumor effect of PAP-1, we cannot exclude that the *in vivo* efficacy is a sum of the effects of PAP-1 on all the parenchymal and peripheral cells expressing Kv1.3. This point could be better clarified in future studies by the use of cell-specific conditional Kv1.3-KO mice.

Taken together, our data suggest that Kv1.3 channels have crucial roles in modifying glial cell functions, modulating the toxic effects of glioma in brain parenchyma and tumor cell infiltration, thus representing candidate targets to re-educated the brain microenvironment in glioma.

## Methods

### Murine primary cultures

Primary neuronal cultures were obtained from p0-p2 C57BL/6N mice as previously described^32^; *α*-tubulin III staining indicates 90% of neurons in the cultures^32^.

Mixed neuronal/glial cultures were prepared from 0–2-day-old (p0–p2) C57BL/6N mice as already described^33^. The ratio of neurons/astrocytes/microglia obtained with this method is respectively 60/35/5%^33^.

Glial cultures were obtained as above^33^; microglial and astrocytic cell populations had less than 2% of contamination, as verified by staining with GFAP and Iba-1^33^.

### Animals and cells

*In vivo* experiments were approved by the Italian Ministry of Health, in accordance with the ethical guidelines on use of animals from the EC Council Directive 2010/63/EU. We used C57BL/6N mice, CX3CR1^gfp/+^ mice, human (GL-15), and murine glioma (GL261) and endothelioma (bEnd.3) cell lines. Primary human GBM cells (GBM18) were obtained from tumor *specimen* from patients who gave written informed consent to the research proposals. Histo-pathological typing and tumor grading were done according to the WHO criteria resulting as grade IV. Tissue was processed as already described^65^. All cells were cultured in DMEM supplemented with 10–20% heat-inactivated FBS, 100 IU/ml penicillin G, 100 μg/ml streptomycin, 2.5 μg/ml amphotericin B and grown at 37 °C in a 5% CO_2_ and humidified atmosphere.

### Neuronal viability assay

Cortical and mixed neuronal cultures at 9–11 DIV were co-cultured with GL261 cells (5 × 10^4^/well) in the presence or absence of PAP-1 (50 nM) or/and DHK (500 μM). After 18 h, cells were treated with detergent-containing buffer and counted in a haemocytometer as already described^33^. In such experiments, empty or clodronate-filled liposomes were given 48 h before starting GL261 co-culture, to specifically deplete microglia from cultures (99.6 ± 0.2%)^33^.

### Patch-Clamp on astrocytes

Astrocytes were plated on coverslips (2 × 10^4^/cm^2^) and tested after 48 h. The whole-cell configuration was used for electrophysiological recordings from astrocytes. Currents and voltages were amplified with a HEKA EPC-10 amplifier (List Medical, Darmstadt, Germany), digitized with a 12 bit A/D converter (TL-1, DMA interface; Axon Instruments, Foster City, CA, USA), and analyzed with the Patch Master package (version 2X60, ELEKTRONIK) and Microcal Origin 6.0 software. For on-line data collection, macroscopic currents were filtered at 3 kHz and sampled at 50 µs/point. The external solution (NES) contained (in mM): NaCl 140, KCl 5, CaCl_2_ 2, MgCl_2_ 2, MOPS 5, glucose 10, (pH 7.4). The internal solution contained (in mM): KCl 150, EGTA-K 1, MgCl_2_ 1, MOPS 5, Na_2_ATP 5 (pH 7.20). Access resistances ranging between 5 and 15 MΩ were achieved and were actively compensated to 50%. Whole-cell patch-clamp recordings in slices (250 μm) obtained from GL261-bearing CX3CR1^gfp/+^ mice were performed by using a Multiclamp 700B amplifier (Molecular Devices, USA). Astrocytes were recognized based on physiological criteria. When 10 mV depolarizing voltage steps were applied from −70 mV to +50 mV all astrocytes displayed large voltage independent currents and a linear current/voltage relationship. 30% of cells displayed also slowly activating voltage-dependent currents. The bath solution (ACSF) contained (in mM): 125 NaCl, 2 KCl, 1.2 MgCl_2_, 2 CaCl_2_, 25 NaHCO_3_, 1.2 NaH_2_PO_4_ and 10 glucose, pH 7.4, 300–305 mOms. ACSF was perfused at a rate of approximately 2 ml/min by using a gravity-driven perfusion system. Glass electrodes (5–7 MΩ) were pulled with a vertical puller (PC-10, Narishige). Pipettes were filled with 140 KCl, 13.4 NaCl, 0.5 CaCl_2_, 1 MgCl_2_, 5 EGTA, 10 Hepes, and 3 Mg-ATP, 0.3 Na3-GTP (280–285 mOsm, pH 7.2). Signals were acquired (sampling 10 kHz, low-pass filtered 2 kHz) with DigiData-1440A using pCLAMP-v10 software (Molecular Devices, USA). Membrane capacitance was estimated as the total charge (i.e., the current integral, Qstep) mobilized in each cell by a 10 mV hyperpolarizing step (Vstep): Qstep/Vstep. The resting membrane potential was recorded in current clamp mode. R_input_ was measured from responses at resting potential to step current injections of amplitude −50 pA to 50 pA in 10 pA increments.

### *In vivo* experiments

Eight-week-old male C57BL/6 N mice were stereotaxically injected with GL261 as previously described^20^. After 7 days, mice were intra-peritoneally (i.p.) treated with PAP-1 (40 mg/kg/die), DHK (10 mg/kg/die) or vehicle [Miglyol (Cremer) and/or PBS]. After 17 days from GL261 injection, animals were sacrificed and tumor volume was evaluated with hematoxylin-eosin staining as previously described^20^. Alternatively, mice were deeply anesthetized and CD11b^+^ cells were immunomagnetically isolated as already described^20^. Obtained cells were lysed in Trizol reagent (Invitrogen, Milan, Italy) for RNA extraction and Real Time PCR (RT-PCR) analysis.

### Brain concentration of PAP-1

Mice treated for 10 days with PAP-1 or vehicle (after 2 and 12 h from the i.p.) were perfused with PBS and sacrificed. Brains were removed and prepared as indicated in Supplementary Methods.

### pH measurements

Astrocytic cultures, plated on coverslips, were loaded with BCECF-AM (10 μM) in NES for 45 min at 37 °C. Time-lapse fluorescence determinations were acquired at room temperature (RT) using a customized digital imaging microscope. Excitation of BCECF was achieved using a Cairn Research – OptoScan monochromator. Fluorescence was visualized using an upright microscope (Olympus) equipped with a 40x water-immersion objective and a digital 14 bit CCD camera system (Cool SNAP MYO, Photometrics). All the peripheral hardware controls, image acquisition and processing were achieved using Metafluor software (Molecular Device). A Glutamate puff was applied with a Pneumo pump (WPI). Changes in BCECF fluorescence distribution were monitored by acquiring images every s (excitation wavelength 490 nm). At each time point the fluorescence increase in the area was calculated as ΔF = F − F0, and then divided for F0 (ΔF/F0, where F0 is the average fluorescence before Glutamate puff).

### Calcium imaging

For measurement of spontaneous calcium oscillations astrocytes were plated onto 35 mm Petri dishes at a density of approximately 1.5 × 10^4^ cells/cm^2^ and experiments were performed at 4–5 days after plating. Petri dishes were loaded with Fluo4 AM (5μM, Molecular probes) for 30 min in NES at 37 °C. Excitation wavelength was 488 nm and sampling rate was 0.5 Hz for 360 s; PAP-1 (100 nM) was added to the bath solution during the acquisition. The fluorescence increase in the area was calculated as ΔF/F0, where F0 is the basal average fluorescence in absence of Ca^2+^ elevations. Only the first peak in each region of interest (ROI) acquired was considered for the amplitude analysis. The frequency was measured by counting the number of Ca^2+^ events occurring during the full acquisition in each ROI.

### D-[^3^H] Aspartate uptake

The uptake of D-[^3^H] Asp was evaluated as previously reported^66^. Astrocytes were incubated with D-[^3^H] Asp (1 μM) and D-Asp (100 μM, for time variable from 2 to 45 min, at 37 °C) with or without PAP-1 (50 nM), DHK (500 μM) or both; samples were counted for radioactivity determination and evaluated for protein content by BCA assay. The uptake rate for each well was expressed as pCi of D-[^3^H] Asp per μg of protein. Data obtained in Na^+^-free buffer (prepared by replacing NaCl with choline chloride) were subtracted for each time points in all experimental conditions.

### Immunofluorescence

Coronal brain sections (20 μm) were washed in PBS, blocked (3% goat serum in 0.3% Triton X-100) for 1 h at RT and incubated overnight at 4 °C with specific antibodies, GFAP (1:750 –Novus Biologicals, NB300-141), Iba1 (1:750 - Wako, 019-19741), GLT1 (1:1000 – AbCam, ab41621), Kv1.3 (1:100 – Alomone Lab, AGP-005). Brain slices were stained with the fluorophore-conjugated secondary antibodies for 1 h at RT and Hoechst for nuclei visualization and analyzed using a fluorescence microscope. For Fluoro-Jade-C staining, we followed the manufacturer instructions (Millipore, AG325).

### Immunoprecipitation and Western blot analysis

Astrocytes (1 × 10^6^ cells) lysate was pre-cleared (with Protein G-Sepharose (Sigma Aldrich), 30 min, 4 °C) and incubated with anti-Sumo1 antibody (10 μg, Santa Cruz, sc-5308) or control IgG (1 h, 4 °C). Samples were immunoprecipitated with Protein G-Sepharose beads (50 μl). After 3 h incubation at 4 °C, beads were washed three times with ice cold buffer, and immunoprecipitated proteins were separated on 7.5% SDS-polyacrylamide gel and visualized by Western blot staining with anti-GLT1 antibody (1:1000, AbCam, ab41621). Protein detection was performed through the chemiluminescence assay Immun-Star Western C Kit (Bio-Rad). Densitometric analysis was carried out with Quantity One software (Bio-Rad).

### Trans Endothelial Electrical Resistance (TEER)

Primary murine astrocytes and bEnd.3 cells were grown to confluence, respectively, on the bottom and upper side of fibronectin-coated membranes in a transwell system with 0.4 μm pores. After three days GL261 cells were then plated in the bottom well of a multiwell plate where the transwell was inserted. TEER was measured using the Millicell® ERS-2 Voltohmmeter (Millipore, Billerica, MA) using chopstick electrodes. TEER values (as Ωcm^2^) were calculated subtracting the blank insert resistances (no cells) from sample resistances, normalized to the support area (in cm^2^).

### Real Time PCR

RNAs extracted from all samples were quantified and retro-transcripted using IScriptTM Reverse Transcription Supermix (Biorad, Italy). Real time PCR (RT-PCR) was carried out in a I-Cycler IQ Multicolor RT- PCR Detection System (Biorad) using SsoFast Eva Green Supermix (Biorad). The PCR protocol consisted of 40 cycles of denaturation at 95 °C for 30 s and annealing/extension at 58 °C for 30 s. The Ct values from each gene were normalized to the Ct value of GAPDH. Relative quantification was performed using the 2^−ΔΔCt^ method and expressed as fold increase. Primer sequences are listed in Supplementary Table 1.

### Phagocytosis assay

NCM- and GCM-treated microglia were treated with PAP-1 (50 nM, 24 h). Red fluorescent FluoSpheres (0.03%, Invitrogen) were added for 1 h and the number of spheres per cell was counted.

### Chemotaxis assays

NCM- and GCM-treated microglia, GL261, GL-15 and GBM 18 cells were incubated 3 h at 37 °C in Boyden chamber with or without PAP-1 (50 nM). Cells adhering to the upper side of the membrane were scraped off, whilst migrated cells were stained with brilliant blue R 250 (Sigma-Aldrich) and counted in more than 20 fields with a 20x objective.

### Form Factor calculation

NCM- and GCM-treated microglia were seeded on glass coverslips, with or without PAP-1 for 24 h, fixed and stained with Alexa-Fluor 488 Phalloidin (Invitrogen) for 20 min together with Hoechst. Fluorescent images were processed using MetaMorph 7.6.5.0 software (Molecular Device, USA) and Form Factor was calculated according the formula 4π area/perimeter^2^.

### Nitrite assay

NO production by microglia cultures was assessed by measuring nitrite accumulation in the culture medium by Griess Reagent Kit according to manufacturer’s instructions (Molecular Probes, MA, USA). The absorbance was measured at 570 nm in a spectrophotometer microplate reader (BioTek Instruments Inc, VT, USA).

### Patch-Clamp on GL261

GL261 cells were patched in the whole-cell configuration. Micropipettes (4–5 MΩ) were filled with a solution containing the following composition (in mM): KCl 135, BAPTA 5, MgCl_2_ 2, HEPES 10, and Mg-ATP_2_ (pH 7.3 adjusted with KOH, osmolarity 290 mOsm; Sigma Aldrich). Voltage-clamp recordings were performed using an Axopatch 200B amplifier (Molecular Devices). Currents were filtered at 2 kHz, digitized (10 kHz) and collected using Clampex 10.2 (Molecular Devices); the analysis was performed off-line using Clampfit 10 (Molecular Devices). The current/voltage (I/V) relationship of each cell was determined applying voltage ramps from −120 to +50 mV for 50 ms holding the cell at −70 mV. Resting membrane potential and membrane capacitance were measured at start of recording. Outward rectifier K^+^ current amplitude was evaluated after subtraction of the leak current by a linear fit of the I/V curve between −100 and −50 mV. Cells were considered as expressing the outward rectifier K^+^ current when the I/V relationship showed a rectification above −30 mV and the amplitude measured at 0 mV was at least 10 pA, after leak subtraction. PAP-1 was bath applied via a valve controlled solution exchanger (VC6 – Warner Instruments).

### Statistical analysis

All statistical analyses were done using SigmaPlot 11.0 Software.

## Electronic supplementary material

Supplementary Information
