# Supplementary material for: Kv1.3 activity perturbs the homeostatic properties of astrocytes in glioma
Source: Sci Rep. 2018 May 16;8:7654. doi: 10.1038/s41598-018-25940-5 (PMC5955950; doi:10.1038/s41598-018-25940-5)
Supplement: Supplementary file 1 — Supplementary Information [file 41598_2018_25940_MOESM1_ESM.pdf]

Kv1.3 activity perturbs the homeostatic properties of astrocytes in glioma

Running Title: Kv1.3 channel involvement in maintaining brain homeostasis.

Alfonso Grimaldi[#1], Giuseppina D'Alessandro[#2], Maria Amalia Di Castro[3], Clotilde Lauro[3], Vikrant Singh[4], Francesca Pagani[1], Luigi Sforna[5], Francesca Grassi[3], Silvia Di Angelantonio[1,3], Luigi Catacuzzeno[6], Heike Wulff[4], Cristina Limatola[2,7,*], Myriam Catalano[2,3]

[#]equally contributing authors

*Correspondence to: cristina.limatola@uniroma1.it

[1]Center for Life Nanoscience – Istituto Italiano di Tecnologia@Sapienza, Viale Regina Elena, 291 - 00185 Rome, Italy.

[2]IRCCS Neuromed, Via Atinense 18, 86077 Pozzilli, IS, Italy.

[3] Department of Physiology and Pharmacology, Sapienza University, Piazzale Aldo Moro, 5 - 00185 Rome, Italy

[4]Department of Pharmacology, University of California Davis, 451 Health Sciences Drive, Davis, CA 95616, USA.

[5]Department of Experimental Medicine, Section of Physiology and Biochemistry, University of Perugia, Piazzale Gambuli - 06123 Perugia, Italy.

[6]Department of Chemistry Biology and Biotechnology, University of Perugia, Via Elce di sotto, 8 - 06123 Perugia, Italy.

[7]Department of Physiology and Pharmacology, Sapienza University, Laboratory affiliated to Istituto Pasteur Italia – Fondazione Cenci Bolognetti, Piazzale Aldo Moro 5 - 00185 Rome, Italy.

Supplementary Methods.

Brain concentration of PAP-1. Mice brains were removed and prepared as follows: 4.0 mL of acetonitrile was added to 200 mg of brain sample and it was homogenized thoroughly using a T25 digital ULTRA-TURRAX® homogenizer (IKA® Works Inc., NC, USA). The homogenized samples were centrifuged for 10 min at 4000 rpm, the supernatant was separated and evaporated under constant air flow. The residues were reconstituted in 200 μL acetonitrile, loaded onto preconditioned SPE cartridges (Hypersep C18, 100 mg, 1 mL, ThermoFisher), washed successively with 1 mL each of 20% and 40% acetonitrile in water, eluted with 2 mL of acetonitrile, evaporated to dryness, reconstituted with 200 μL. *LC/MS analysis:* LC/MS analysis was performed with a Waters Acquity UPLC (Waters, NY, USA) equipped with a Acquity UPLC BEH 1.7μM C-18 column (Waters, New York, NY) interfaced to a TSQ Quantum Access Max mass spectrometer (MS) (Thermo Fisher Scientific, Waltham, MA, USA). The isocratic mobile phase consisted of 80% acetonitrile and 20% water, both containing 0.1% formic acid with a flow rate of 0.25 ml per minute. Under these conditions PAP-1 had a retention time of 1.05 min. Using Heated electrospray ionization source (HESI II) in positive ion mode, capillary temperature 270 °C, vaporizer temperature: 350 °C, spray voltage 3000 V, sheath gas pressure (N_2) 30 units, PAP-1 was analyzed by the selective reaction monitoring (SRM) transition of its molecular ion peak 351.16 (M+1) into 107.19 *m/z.* A 10-point calibration curve from 5nM to 10 μM was developed for quantification.

Supplementary Table 1.

Gene	**Primer Forward (5'-3')**	**Primer Reverse (5'-3')**
Arg1	CTCCAAGCCAAAGTCCTTAGAG	AGGAGCTGTCATTAGGGACATC
CD86	AGAACTTACGGAAGCACCCA	GGCAGATATGCAGTCCCATT
CD163	GCTAGACGAAGTCATCTGCACTGGG	TCAGCCTCAGAGACATGAACTCGG
CD206	CAAGGAAGGTTGGCATTTGT	CCTTTCAGTCCTTTGCAAGT
Claudin-5	TAAGGCACGGGTAGCACTCA	GCCCAGCTCGTACTTCTGTG
Fizz1	CCAATCCAGCTAACTATCCCTCC	ACCCAGTAGCAGTCATCCCA
Occludin	ATCCTGTCTATGCTCATTATTGTG	CTGCTCTTGGGTCTGTATATCC
Gapdh	TCGTCCCGTAGACAAAATGG	TTGAGGTCAATGAAGGGGTC
IL1β	GCAACTGTTCCTGAACTCAACT	ATCTTTTGGGGTCCGTCAACT
IL15	CATCCATCTCGTGCTACTTGTGTT	CATCTATCCAGTTGGCCTCTGTTT
iNOS	ACATCGACCCGTCCACAGTAT	CAGAGGGGTAGGCTTGTCTC
TNFα	GTGGAACTGGCAGAAGAG	CCATAGAACTGATGAGAGG
Ym1	CAGGTCTGGCAATTCTTCTGAA	GTCTTGCTCATGTGTGTAAGTGA
Zo-1	GGAGCTACGCTTGCCACACT	GGTCAATCAGGACAGAAACACAGT

Supplementary Table 1. List of primers used for Real Time PCR experiments.

Supplementary Figures.

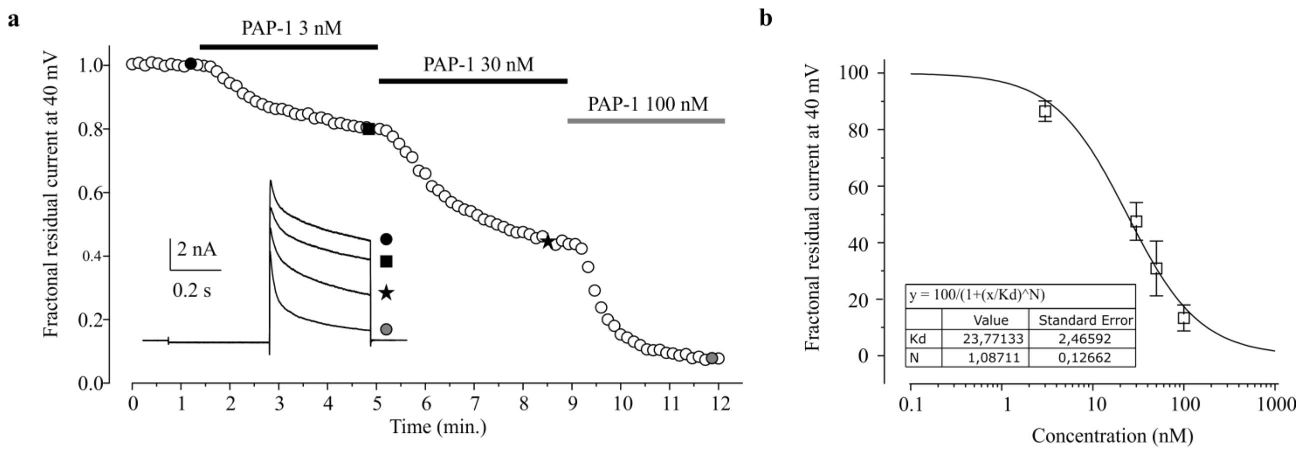

Supplementary Figure 1. Dose-response relationship for the inhibition of the sustained voltage-gated component by PAP-1.a) Time course of the current evoked by a 40 mV voltage step, preceded by a -110 mV prepulse in a cell were 3, 30, and 100 nM PAP-1 were sequentially applied. The current was measured at the end of the depolarizing pulse. Inset: representative current traces taken under control conditions, and in presence of different PAP-1 concentrations.b) Plot of the mean fractional residual current as a function of the PAP-1 concentration. The solid curve represents the best fit of the experimental data with a Hill relationship. The best fit parameters were Kd=23.8 nM, n_H=1.09.

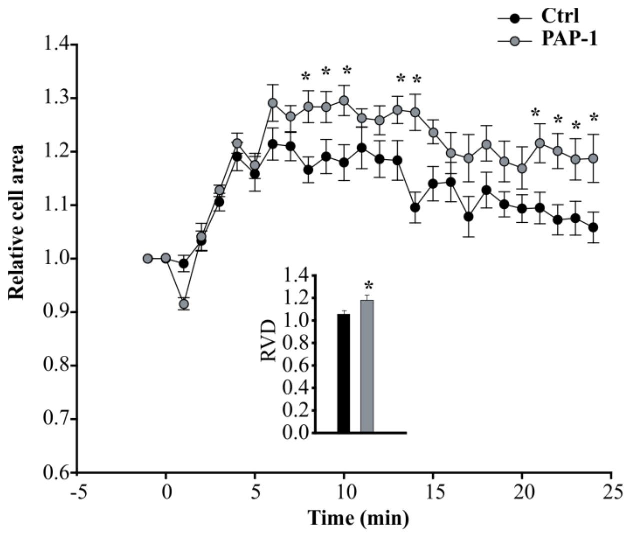

Supplementary Figure 2. Kv1.3 inhibition impairs regulatory volume decrease (RVD) of GL261 cells. Cell area changes in time lapse recordings of GL261 cells untreated (Ctrl) or PAP-1 (50 nM) treated, challenged with hypo-osmotic solution (50% H_2O added to Locke solution, 157 mOsm), at indicated time points. In the inset regulatory volume decreased at 25 min. n=20, *p=0.023, unpaired *t*-test.
